# Gut microbiota is a potential factor in shaping phenotypic variation in larvae and adults of female bumble bees

**DOI:** 10.3389/fmicb.2023.1117077

**Published:** 2023-03-01

**Authors:** Baodi Guo, Jiao Tang, Guiling Ding, Shibonage K. Mashilingi, Jiaxing Huang, Jiandong An

**Affiliations:** ^1^Key Laboratory for Insect-Pollinator Biology of the Ministry of Agriculture and Rural Affairs, Institute of Apicultural Research, Chinese Academy of Agricultural Sciences, Beijing, China; ^2^Department of Crop Sciences and Beekeeping Technology, College of Agriculture and Food Technology, University of Dar es Salaam, Dar es Salaam, Tanzania

**Keywords:** *Bombus terrestris*, worker, queen, development, sociality

## Abstract

Host symbionts are often considered an essential part of the host phenotype, influencing host growth and development. Bumble bee is an ideal model for investigating the relationship between microbiota and phenotypes. Variations in life history across bumble bees may influence the community composition of gut microbiota, which in turn influences phenotypes. In this study, we explored gut microbiota from four development stages (early-instar larvae, 1st instar; mid-instar larvae, 5th instar; late-instar larvae, 9th instar; and adults) of workers and queens in the bumble bee *Bombus terrestris* using the full-length 16S rRNA sequencing technology. The results showed that morphological indices (weight and head capsule) were significantly different between workers and queens from 5th instar larvae (*p* < 0.01). The alpha and beta diversities of gut microbiota were similar between workers and queens in two groups: early instar and mid instar larvae. However, the alpha diversity was significantly different in late instar larvae or adults. The relative abundance of three main phyla of bacteria (Cyanobacteria, Proteobacteria, and Firmicutes) and two genera (*Snodgrassella* and *Lactobacillus*) were significantly different (*p* < 0.01) between workers and queens in late instar larvae or adults. Also, we found that age significantly affected the microbial alpha diversity as the Shannon and ASVs indices differed significantly among the four development stages. Our study suggests that the 5th instar larval stage can be used to judge the morphology of workers or queens in bumble bees. The key microbes differing in phenotypes may be involved in regulating phenotypic variations.

## Introduction

Microbial symbionts are often considered an important part of the host phenotype, participating in host health maintenance, nutrition uptake, energy release, and regulation of host physiology (Tremaroli and Bäckhed, 2012; Archie and Tung, 2015). Similarly, gut bacterial communities reflect changes in host phenotype and are influenced by the host’s diet and physiology (Chandler et al., 2011; Koch and Schmid-Hempel, 2011). Whether phenotypic diversity among individuals results from host–microbe interactions deserves further exploration.

Compared with the gut microbiota of many other animals, the social bumble bees and honey bees harbor a relatively simple yet specialized gut microbiota, including *Snodgrassella alvi*, *Lactobacillus,* and *Gilliamella apicola* (Neveling et al., 2012; Kwong and Moran, 2013; Zheng et al., 2016). It is reported that these microbes have many beneficial interactions with bumble bees and honey bees, including increasing metabolic function and protection from invading pathogens (Kwong et al., 2017).

The eusocial Hymenoptera provides an excellent opportunity to explore the relationship between microbiota and host phenotypic variation. In many social species of ants, bees, and wasps, individuals in the same colony show differences in the division of labor, accompanied by changes in nutritional status and physiology (Wilson, 1971). For example, in bumble bees, queens lay fertilized eggs (one set of chromosomes from the drone, one from the queen) that mature into workers and new queens; Alaux et al., 2007). Morphology, physiology, behavior, longevity, and other life-history traits significantly differ between queens and workers, although they are derived from the same genome (Weiner and Toth, 2012). Compared to workers, the queen is larger with more developed reproductive organs and a longer life span (Bloch and Hefetz, 1999). Also, since they share a nested environment and transfer food using trophallaxis during development, the larvae of workers and queens have a similar diet (Pereboom et al., 2003).

Shared social communities and environments generally contribute to common gut microbiota (Degnan et al., 2012; Zhang and Zheng, 2022). In support of this view, the gut microbiota sampled from social species of bees, such as honey bees and bumble bees, tend to be host-specific (Koch et al., 2013). Similar gut microbiota has also remarkably been observed for workers from different colonies of honey bees (Martinson et al., 2011). The physical interactions in social species create a potential for colony-wide transmission of gut microbiota, suggesting little microbiome variation among individual members in a colony, such as between soldiers and workers in termites (Otani et al., 2019).

However, some gut microbiotas may be involved in phenotypic variation by improving digestion and enhancing metabolism (Engel and Moran, 2013). These gut microbiota characteristics in turn correspond to their roles in the division of labor (Engel et al., 2012). Specifically, workers harbor more complex gut communities than queens, presumably more suited to process food than queens. In *Apis mellifera*, queen microbiomes may enhance the metabolic conversion of energy from food to egg production (Aupinel et al., 2005). Within the worker caste, young (nurse) honey bees that perform tasks inside the hive, such as brood care, have more diverse gut microbial communities than foragers (Jones et al., 2018). Furthermore, age has an influence on gut microbial communities during the development of bees (Hroncova et al., 2019). For example, the genera *Bartonella* and *Enterobacter* are mainly founded in the first instar larvae, while *Acinetobacter* and *Rhodococcus* are mainly founded in the fifth instar larvae of the bumble bee (*Andrena camellia*; Kou et al., 2022).

*Bombus terrestris*, one of the eusocial Hymenoptera species, is an ideal model to investigate the relationship between microbiota and phenotypes (Koubová et al., 2019). Although the diet of queens and workers is similar, the gut microbial composition may differ. In this study, we first measured morphological changes between workers and queens during larval development. Then, we explored changes in gut microbiota in larvae and adults of bumble bee workers and queens. We hypothesized that (i) the richness of some key microbiotas is different between workers and queens, (ii) the diversity of microbiota changes with the development of bumble bees. The findings of this study will improve our understanding of possible relationships between gut microbial communities and the phenotypic variation of bumble bees.

## Materials and methods

### Sample collection

*Bombus terrestris* colonies were reared under standard laboratory conditions (27 ± 2°C, 60 ± 5% relative humidity; Gurel and Karsli, 2013). Fresh pollen and sugar solutions (1:1, w/v) were provided *ad libitum* as a diet (Zhang et al., 2021).

To collect the worker and queen larvae accurately, we monitored the whole development period of the larvae. Monitoring began when the colonies had about 100 workers, which is close to the time for new queens to develop. At this stage, we recorded the location and time of each batch of eggs laid by queens daily, then took one larva on the 1st, 5th, and 9th, whereas the remaining were left to develop into adults. We determined whether the collected larvae were queens or workers based on the adult bees that emerged from the remaining larvae. Moreover, to explore the development of the queens and workers throughout the larval span, we took the larvae of each instar of workers and queens to determine the morphological index. The larval body weight was measured using a digital electronic scale (accurate to within 0.1 mg) (BSA124S, Sartorios, Gottingen, Germany), and the head capsule was observed using a microscope (SZ2ILST, Olympus Corporation, Tokyo, Japan; Cnaani et al., 1997).

**Figure 1:**
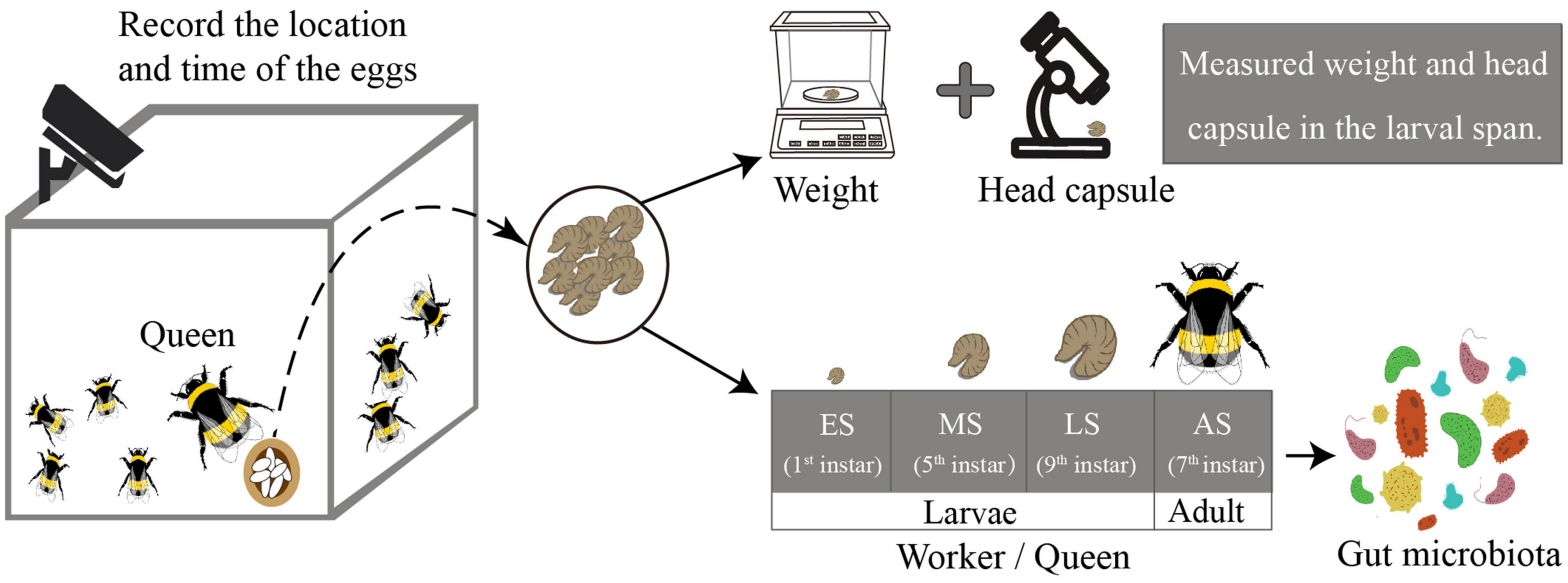
Schematic overview of this study. We monitored the development of larval in *Bombus terrestris*. Some larvae were used to measure morphological indicators while others were used to determine microbiota. The gut microbiota was sampled at four stages (ES represents early larvae stage, MS represents mid larvae stage, LS represents late larvae stage, and AS represents adult stage).

Gut microbiota was sampled from four stages, three of which were larval stages and one was the adult bumble bee stage (Figure 1). These samples consisted of: (a) early-instar queen-destined larvae (EQ, 1 instar larvae, 80 larvae from six colonies, representing four biological replicates) and early-instar worker-destined larvae (EW, 1 instar larvae, 80 larvae from six colonies, representing four biological replicates); (b) mid-instar queen-destined larvae (MQ, 5 instar larvae, 32 larvae from six colonies, representing eight biological replicates) and mid-instar worker-destined larvae (MW, 5 instar larvae, 20 larvae from six colonies, representing 5 biological replicates); (c) late-instar queen-destined larvae (LQ, 9 instar larvae, 8 larvae from 6 colonies, representing 8 biological replicates), and late-instar worker-destined larvae (LW, 9 instar larvae, 8 larvae from 6 colonies, representing 8 biological replicates); (d) adult queens (AQ, 7 instar adults, 8 individuals from 6 colonies, representing 8 biological replicates), and adult workers (AW, 7 instar adults, 8 individuals from 6 colonies, representing 8 biological replicates). Harvested samples were kept at –80°C until the total 16S rRNA was extracted.

### DNA extraction and sequencing of 16S rRNA

Following the manufacturer’s instructions, DNA from fecal contents was extracted by a QIAamp DNA Stool Mini Kit from Qiagen (Germany). The primer sequences of the full-length 16S rRNA were as follows: 27F (5’-AGAGTTTGATCMTGGCTCAG-3’) and 1492R (5’-ACCTTGTTACGACTT3’; He et al., 2020). The PCR reaction conditions were 95°C for 5 min, 95°C for 30 s, 50°C for 30 s, and 72°C for 1 min, with 25 cycles in a reaction volume of 10 μl. PCR products were purified by adding Genome DNA Clean magnetic beads of equal volume. DNA concentration was measured using Qubit® DNA Assay Kit in Qubit® 2.0 Fluorometer (Life Technologies, CA, United States). The samples with DNA concentrations greater than 20 ng/μL were used for constructing the DNA library (Pacific Biosciences SMRT bellTM Template Prep Kit 1.0), sequencing by PacBio SMRT RS II instrument P6 - C4 reagent computer, and the movie times were 240 min by CCS mode. Demultiplexed CCSs were generated using the RS_ReadsOfInsert.2.1 protocol in SMRT Analysis software version 2.3.0 with the following settings: Minimum Full Passes = 2, Minimum Predicted Accuracy = 90, and Minimum Barcode Score = 22 in Symmetric Barcode Mode (Callahan et al., 2019). All subsequent library building and sequencing work were conducted at Berry Genomics Biotech Co., Ltd. (China).

### Data processing of gut samples

The data from different samples were identified according to the barcode sequence and converted into fastq datasets. We used the DADA2 pipeline within the QIIME2 (version 2021.8) package^1^ to filter low-quality and chimera errors and generate unique sequence variants. Because the “operational taxonomic units (OTUs)” resulting from DADA2 are created through the grouping of unique sequences, these sequences are the equivalent of 100% OTUs, and are generally referred to as amplicon sequence variants (ASVs; Straub et al., 2020). The obtained ASVs were taxonomically annotated in the Greengene reference database (Smith et al., 2020).

### Statistical analysis

The alpha diversity measures such as the observed ASVs (i.e., the total number of ASVs detected per sample) and Shannon index (i.e., the number of taxa and evenness of their distribution, more influenced by the richness and rare species) were compared (Delbeke et al., 2022). The Kruskal–Wallis test was used to compare the differences among groups as some of the variables were not normally distributed. The effects of age and phenotype in the 2-by 2-factor design on alpha diversity were analyzed by linear mixed model in R (implemented in R package limerTest; Bates and Pinheiro, 1998).

Bray–Curtis dissimilarity was used to visualize beta diversity to examine the difference in microbial composition among the sampled groups. The principal component analysis (PCA) was visualized in R (implemented in R package vegan) (Segata et al., 2011). We used pairwise permutation multivariate analysis of variance (PERMANOVA) with 999 random permutations to test the significance of the differences among groups (Anderson, 2001).

Also, we used PICRUSt2 to predict the functional capacity of the gut microbial community. The ASVs table was supplied to PICRUSt2, and then predicted functional genes were categorized into MetaCyc pathways (Low et al., 2021).

## Results

### Fifth-instar is the key point for morphological difference between worker and queen bumble bees

**Figure 2:**
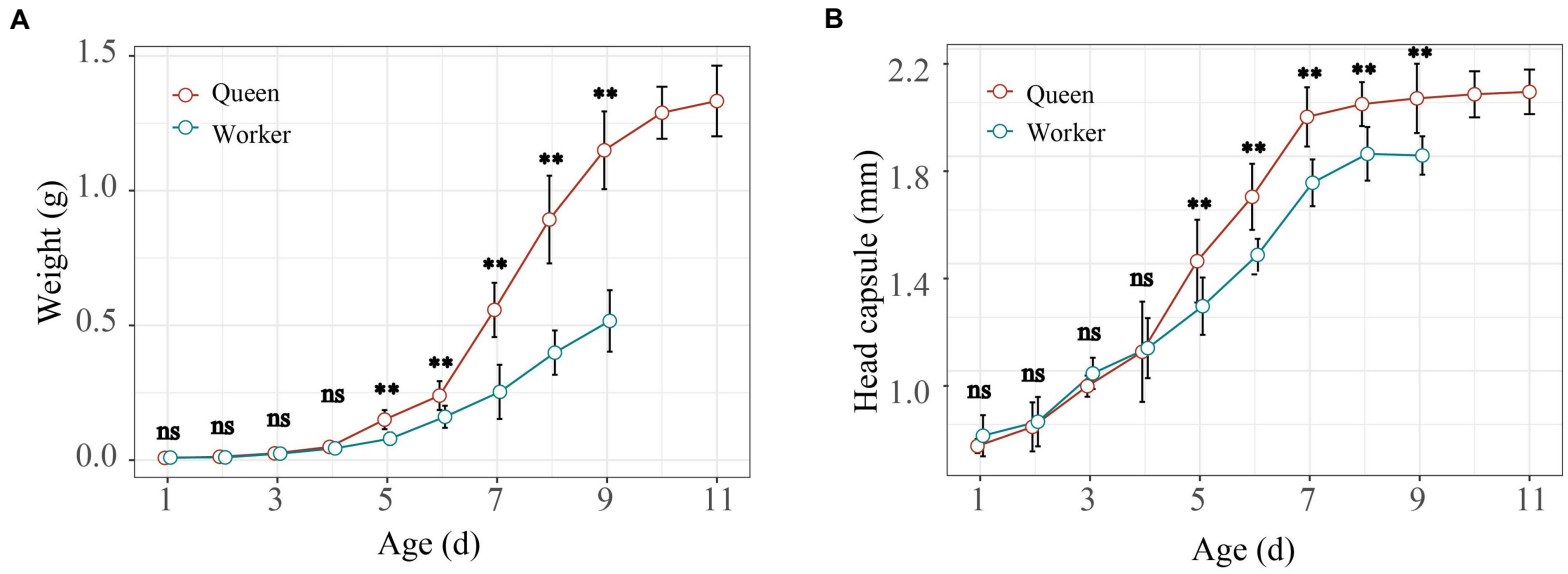
Larval growth and development curve of larvae in *Bombus terrestris*. The weight **(A)** and head capsule **(B)** of worker and queen larvae during their entire development (*t*-test; ns represents *p* > 0.05, * represents *p* < 0.05, ** represents *p* < 0.01).

The whole development period of worker-destined larvae was different from queen-destined larvae. Queen larvae took more time (11 days) than worker larvae (9 days) for development. In addition, significant body weight differences were found between queen and worker bees at 5th - 9th instar larvae (Figure 2A; *t*-test, *p* < 0.01). Similarly, the head capsules were significantly larger in queens than in workers from 5th - 9th instar larvae (Figure 2B; *t*-test, *p* < 0.01), while there were no differences from the 1st – 4th instar larvae (Figure 2B; *t*-test, *p* > 0.05). Thus, our results showed that the 5th instar larval stage was the key period at which the weight and head capsule began to show significant variation between workers and queens of bumble bees.

### Variation of microbial compositions between worker and queen bumble bees

To identify whether gut microbiota exhibited variations in response to workers and queens, we analyzed 16S rRNA sequences from gut samples. After quality control, a total of 267,307 high-quality sequences were retained for all samples and an average of 5,043 sequences were obtained per sample.

**Figure 3:**
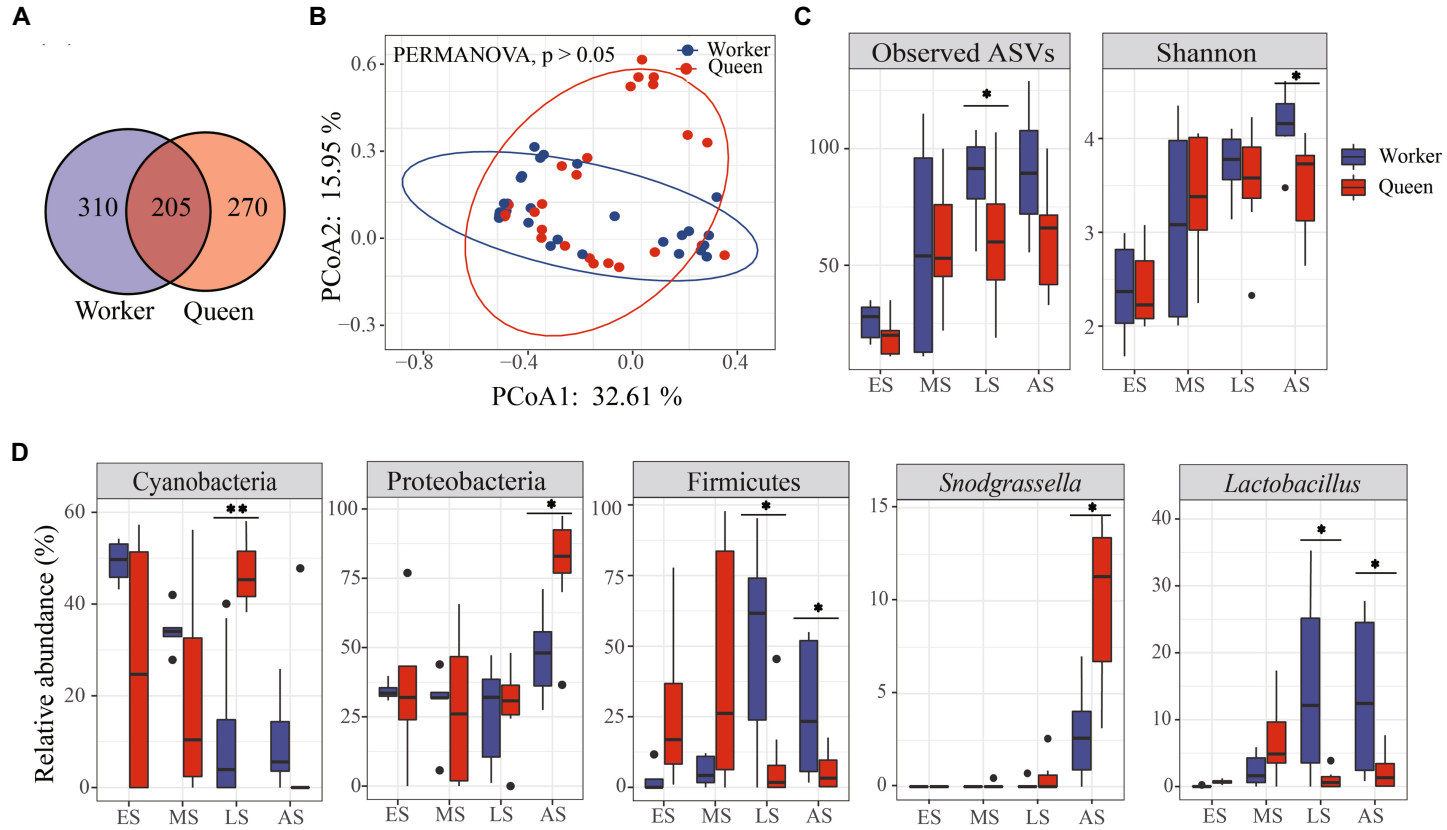
The composition and diversity of microbiota in *Bombus terrestris*. **(A)** Venn diagram of common amplicon sequence variants (ASVs). Circle A represents worker samples; circle B represents queen samples. **(B)** PCoA plot based on Bray-Curtis distance metrics depicting the differences in microbial community structure between worker and queen bumble bees. **(C)** Alpha diversity (ASVs and Shannon indices) of microbiota between workers and queens (Wilcoxon test, * represents *p* < 0.05). **(D)** The relative abundance of phyla and genera significantly differed between workers and queens (Wilcoxon test, * represents *p* < 0.05).

Overall, there was no significant difference in microbial diversity between workers and queens. We found a total of 205 shared ASVs, and 310 and 270 ASVs that were specific to worker and queen samples, respectively (Figure 3A). In addition, the linear mixed-effects model analysis indicated that phenotype could not significantly affect microbial alpha diversity within the bumble bees (Table 1; *F*_ASVs_ = 0.79, *p* = 0.3783; *F*_Shannon_ = 1.99, *p* = 0.1646). Based on PERMANOVA of the Bray-Curtis distance matrix, we revealed that microbial communities were not significantly different between workers and queens (*p* > 0.05). The principal-coordinate analysis (PCoA) graphs clearly illustrated that worker and queen samples were clustered together (Figure 3B).

**Table 1:** Linear mixed-effects model by restricted maximum likelihood (REML) for alpha diversity of ASV and Shannon indices in *Bombus terrestris*.

	d.f.	*F*	Value of *p*
Main effects – ASV			
AIC = 558.685			
Age	50	2.63	0.06
Phenotype	50	0.79	0.3783
Main effects – Shannon			
AIC = 134.9644			
Age	50	4.54	0.0067
Phenotype	50	1.99	0.1646

d.f., degrees of freedom; AIC, Akaike information criterion.

However, the ASVs index was significantly different between workers and queens, indicating significant differences in the microbial richness at the late stage (Figure 3C; Wilcoxon test, *p* < 0.05). The Shannon index was higher in workers than in queens, revealing a significant difference in the abundance of bacteria at the adult stage (Figure 3C; Wilcoxon test, *p* < 0.05). Moreover, we found that the relative abundance of three main phyla (Cyanobacteria, Proteobacteria, and Firmicutes) significantly differed between queens and workers. The relative abundance of Cyanobacteria was significantly higher in queens compared to workers of the late larvae group (Figure 3D; Wilcoxon test, *p* < 0.01) while the relative abundance of Proteobacteria was significantly high in queens than in workers of the adult group (Figure 3D; Wilcoxon test, *p* < 0.05). Firmicutes were significantly less abundant in queens than in workers of both late larvae and adult groups (Figure 3D; Wilcoxon test, *p* < 0.05).

Moreover, the taxonomic composition and distribution of microflora at the genus level revealed that the relative abundance of *Snodgrassella* was significantly higher in queens than in workers of the adult group (Figure 3D; Wilcoxon test, *p* < 0.05). In contrast, the relative abundance of *Lactobacillus* genus was significantly lower in queens than in workers of both late larvae and adult groups (Figure 3D; Wilcoxon test, *p* < 0.05).

### Microbial diversity varied as the age increased in worker and queen bumble bees

**Figure 4:**
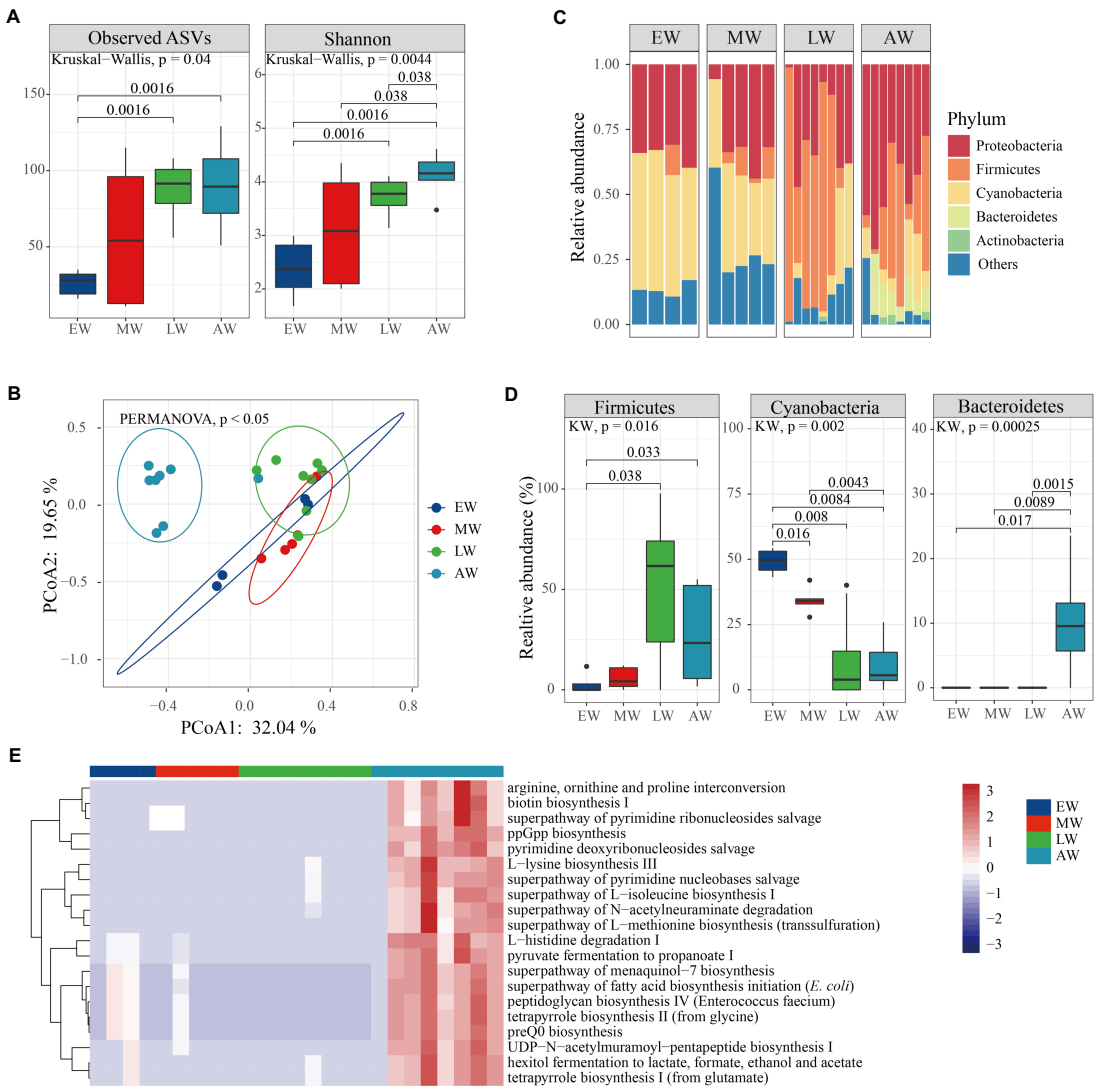
The variation of microbial diversity between the four stages of workers in *Bombus terrestris*. **(A)** Alpha diversity (ASVs and Shannon indices) of microbiota in worker bumble bees using Kruskal–Wallis test. **(B)** PCoA plot based on Bray–Curtis distance metrics depicting the differences in microbial community structure (ES represents early larvae stage, MS represents mid larvae stage, LS represents late larvae stage. AS represents adult stage). **(C)** Phylum-level microbial composition of bumble bees. **(D)** The relative abundance of phyla significantly differed between the four groups (Kruskal–Wallis test, *p* < 0.05). **(E)** The relative abundance of the top 20 metabolic pathways using PICRUSt-predicted Metacyc orthologs.

Linear mixed-effects model analysis indicated that age was an important factor for changes in microbial diversity within bumble bees (Table 1; *F*_ASVs_ = 2.63, *p* = 0.06; *F*_Shannon_ = 4.54, *p* = 0.0067). In worker bumble bees, ASVs and Shannon indices were not significantly different (Figure 4A; Kruskal–Wallis test, *p* = 0.22, *p* = 0.072), but the values were higher in the three late groups (MW, LW, and AW) than in the EW group. Additionally, the PERMANOVA analysis showed that microbial communities (beta-diversity) were significantly different between groups (Figure 3B; *p* < 0.05). The PCoA plot showed that larval samples (EW, MW, and LW) were clustered together, different from adult bumble bee samples (AW; Figure 4B). Furthermore, the relative abundance of the top five phyla (Proteobacteria, Firmicutes, Cyanobacteria, Bacteroidetes, and Actinobacteriota) differed in the four groups (Figure 4C). The relative abundance of Firmicutes was significantly higher in both LW and AW than in EW and MW groups, whereas the relative abundance of Cyanobacteria was significantly lower in LW and AW groups (Figure 4D; Wilcoxon test, *p* < 0.05). The relative abundance of Bacteroidetes was significantly high in the AW than in other groups (Figure 4D; Wilcoxon test, *p* < 0.05). Thus, the diversity of microbiota was affected by the age of workers.

**Figure 5:**
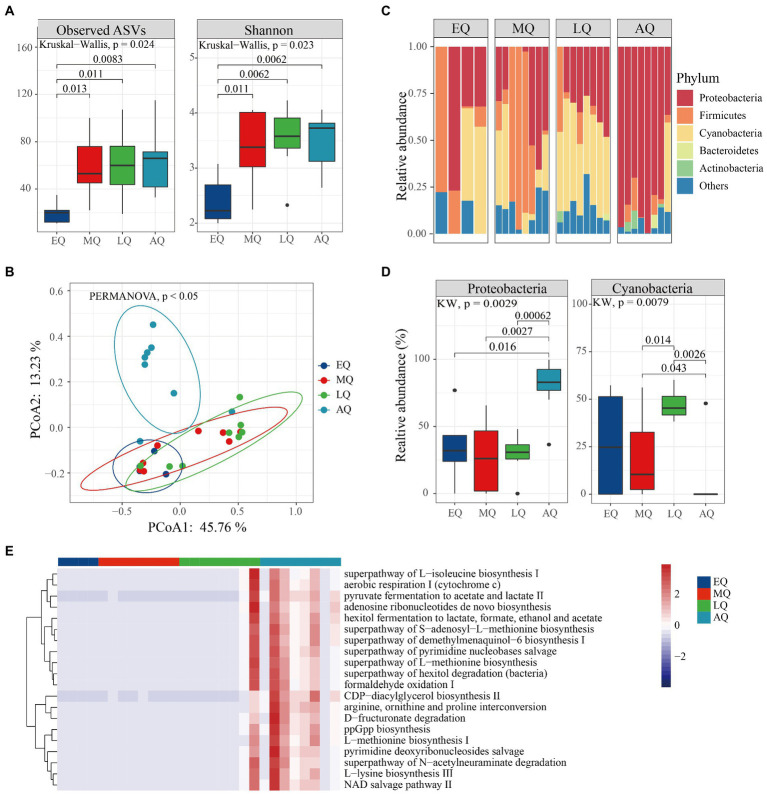
The variation of microbial diversity between four stages of queens in *Bombus terrestris*. **(A)** Alpha diversity (ASVs and Shannon indices) of microbiota in queen bumble bees using Kruskal–Wallis test. **(B)** PCoA plot based on Bray-Curtis distance metrics depicting the differences in microbial community structure (ES represents early larvae stage, MS represents mid larvae stage, LS represents late larvae stage. AS represents adult stage). **(C)** Phylum-level microbial composition of bumble bees. **(D)** The relative abundance of phyla was significantly different among the four groups (Kruskal–Wallis test, *p* < 0.05). **(E)** The relative abundance of the top 20 metabolic pathways using PICRUSt-predicted Metacyc orthologs.

Likewise, in queen bumble bees, we found both ASVs and Shannon indices significantly differed between the four groups (Figure 5A; Kruskal–Wallis test, *p* = 0.024, *p* = 0.023). Both ASVs and Shannon indices were significantly higher in MQ, LQ, and AQ than EQ group (Figure 5A; Wilcoxon test, *p* < 0.05). The PERMANOVA analysis revealed that microbial communities (beta-diversity) were significantly different between the four groups (*p* < 0.05). The PCoA plot showed that larval samples (EQ, MQ, and LQ) were clustered together, which were also different from adult bumble bee samples (AQ; Figure 5B). In addition, the relative abundance of the top five phyla (Proteobacteria, Firmicutes, Cyanobacteria, Bacteroidetes, and Actinobacteriota) differed in the four groups (Figure 5C). Proteobacteria were significantly more abundant in AQ than in other groups (Figure 5D; Wilcoxon test, *p* < 0.05), while Cyanobacteria were significantly less abundant in AQ than in MQ and LQ groups (Figure 5D; Wilcoxon test, *p* < 0.05). Therefore, our results showed that the richness and abundance of microbiota were also influenced by age in queens.

Furthermore, bacterial functions were predicted and ‘mapped’ in the MetaCyc database. A total of 220 metabolic pathways encoded were predicted, 67 and 26 of which were significantly different in queens and workers, respectively (Supplementary Table S1; Kruskal–Wallis test, *p* < 0.01). The worker bacteria-encoded functions that were active throughout the life cycle mainly included amino acid metabolisms and biosynthesis (arginine ornithine and proline interconversion, l-histidine degradation I and l-lysine biosynthesis III; Figure 4E; Kruskal–Wallis test, *p* < 0.01). Also, the queen bacteria-encoded functions also mainly included amino acid biosynthesis (arginine ornithine and proline interconversion, l-methionine biosynthesis I and l-lysine biosynthesis III) and generation of precursor metabolites and energy (hexitol fermentation to lactate, pyruvate fermentation to acetate and lactate II and aerobic respiration I; Figure 5E; Kruskal–Wallis test, *p* < 0.01). Overall, our results revealed that more pathways were enriched at the adult stage than at the larval stages of both worker and queen bumble bees.

## Discussion

The gut microbiota of eusocial Hymenoptera is highly conserved and represented by a few phylotypes (Cornet et al., 2022). Its composition tends to be dominated by phyla Proteobacteria, Firmicutes, Actinobacteria, and Bacteroidetes in *Amdrena camellia* (Kou et al., 2022). Likewise, Proteobacteria and Firmicutes are the most abundant taxa in Pheidole rugaticeps Emery (Hymenoptera: Formicidae) (Ashigar and Ab Majid, 2021). The study found that Proteobacteria, Firmicutes, Cyanobacteria, Bacteroidetes, and Actinobacteria were the main phyla in queen and worker bumble bees. The alpha and beta diversity of these microbial communities was not significantly different between workers and queens (Table 1; Figure 3C). One potential explanation is that *B. terrestris* shares nectar *via* honeypots inside the colony, thus the diversity of microbiota shows similarity (McFrederick et al., 2013).

Gut bacteria increase weight gain in young adult bees, affect the expression of genes governing insulin and vitellogenin levels, and increase sucrose sensitivity (Zheng et al., 2017). Additionally, gut bacteria produce short-chain fatty acids, with acetate and propionate as the major metabolites, as in the guts of humans and other animals (Lee et al., 2015). In our study, significant body weight differences were observed between queen and worker bees at 5th – 9th instar larvae (Figure 2A). The 5th and 9th instar larvae stages showed differences in alpha diversity between worker and queen bees. Also, these instar larvae stages exhibited differences in the relative abundance of bacterial groups, such as Cyanobacteria, Proteobacteria, and Firmicutes (Figures 3C,D), which play an important role in the digestion and absorption of food. Cyanobacteria are photosynthetic prokaryotes that use light energy to split water and transfer electrons to produce ATP in insects (Kang et al., 2021). Proteobacteria can digest secondary metabolites (such as terpenes, alkaloids, glycosides, and phenolic compounds) of insect hosts and help to maintain the growth and development of insects; its absence leads to slower development in insects (Shah et al., 2021). Studies show that Firmicutes play a role in energy absorption in insects (Zhang et al., 2022).

Moreover, we found *Snodgrassella* and *Lactobacillus* significantly differed in worker and queen bumble bees (Figure 3D). *Snodgrassella* is the core genus in honey bees (*Apis* spp.) and bumble bees (*Bombus* spp.) (Cornet et al., 2022). Functional analyses revealed the importance of small proteins, defense mechanisms, amino acid transport, and metabolism in *Snodgrassella* genus (Hammer et al., 2021). Honey bees also associate with *Lactobacillus* from flowers and may therefore obtain these bacteria from flowers (McFrederick et al., 2017). A recent study shows that *Lactobacillus plantarum* influences mate preferences in *Drosophila melanogaster*, possibly through alterations of cuticular hydrocarbon sex pheromones affecting the phenotype (Sharon et al., 2010). Thus, our results elucidate the important role of bacteria in bumble bee developments.

Microbial composition is significantly affected by age during bee development (Tarpy et al., 2015). Linear mixed-effects model analysis indicated that age was the major factor shaping microbial community alpha diversity within bumble bees (Table 1). This is consistent with the findings on *Apis mellifera* that there is an increase in the number of isolated bacterial colonies and diversity as the larvae age (Evans and Armstrong, 2006). One possible explanation is that the absence of defecation during the larval stage contributes to increasing diversity and abundance with successive larval instar. In addition, our results showed that the alpha diversity was lower in early larvae stage but was higher in other stages of bumble bees. Bacterial diversity may also have increased due to the increase in pollen in the larval food (Voulgari-Kokota et al., 2020). When glandular secretions of bees are mixed with increasing amounts of sugary crop contents, the larval food becomes more susceptible to microbial inoculation from pollen grains (Vojvodic et al., 2013).

Finally, microbial communities in the larval gut can differ from those in adults of Hymenoptera (Li et al., 2021). For example, Gammaproteobacteria, Acetobacteraceae, Firmicutes, and *Bacillus* spp. differ between larvae and adult honey bees (Vojvodic et al., 2013). Our results showed that the microbial diversity of larval samples exhibited much higher similarity, different from adult bumble bees (Figures 4B, 5B). The bacteria-encoded functions that were active throughout the life cycle included mainly biosynthesis and generation of precursor metabolites and energy in adults than in larval bumble bees (Figures 4E, 5E). Bacteria play a role in metabolism processing during the early and fragile stages of bumble bees.

## Conclusion

Fertilized eggs produced by the bumble bee queen develop into two phenotypes, the new queen (large size) and the worker (small size). This study explore the relationship between microbiota and phenotypic variation in bumble bees. The results show that alpha and beta diversity of gut microbiota is similar in workers and queens. However, the relative abundance of three main phyla of bacteria (Cyanobacteria, Proteobacteria, and Firmicutes) and two genera (*Snodgrassella* and *Lactobacillus*) are significantly different. Furthermore, results show microbiota diversity is significantly affected by age in bumble bees. The findings of this study give insights for further studies on the relationships between gut microbiota and phenotypic variation in female bumble bees.

## Data availability statement

The datasets presented in this study can be found in online repositories. The names of the repository/repositories and accession number(s) can be found in the article/Supplementary material.

## Author contributions

JA conceived the research project. BG collected the samples, analyzed the data, and sequenced the samples. BG, JT, GD, SM, JH, and JA wrote the manuscript. All authors read and approved the final manuscript.

## Funding

This research was funded by the Natural Science Foundation of China (31672500 and 32072797), the Agricultural Science and Technology Project of Gansu Province (GSLK-2021-17 and GSLK-2022-16), the Special Program for Basic Resources of Science and Technology (2018FY100404), the Agricultural Science and Technology Innovation Program (CAAS-ASTIP-2015-IAR), and the China Agriculture Research System-Bee (NYCYTX-44-KXJ5).

## Conflict of interest

The authors declare that the research was conducted in the absence of any commercial or financial relationships that could be construed as a potential conflict of interest.

## Publisher’s note

All claims expressed in this article are solely those of the authors and do not necessarily represent those of their affiliated organizations, or those of the publisher, the editors and the reviewers. Any product that may be evaluated in this article, or claim that may be made by its manufacturer, is not guaranteed or endorsed by the publisher.

## Footnotes

^1^ https://qiime2.org
